# Characterization of Severe Fever with Thrombocytopenia Syndrome in Rural Regions of Zhejiang, China

**DOI:** 10.1371/journal.pone.0111127

**Published:** 2014-10-30

**Authors:** Lei Zhang, Ling Ye, David M. Ojcius, Xiuyu Lou, Chengwei Wang, Cen feng, Yi Sun, Zhongfa Wang, Shibo Li, Yanjun Zhang

**Affiliations:** 1 Zhejiang Provincial Center for Disease Control and Prevention, Hangzhou, Zhejiang, P.R. China; 2 Department of Civil and Environmental Engineering, University of Alberta, Edmonton, AB, Canada; 3 Center for Disease Control and Prevention of Daishan, Zhoushan, Zhejiang, P.R. China; 4 Health Sciences Research Institute and School of Natural Sciences, University of California Merced, Merced, California, United States of America; 5 Zhoushan Hospital, Zhoushan, Zhejiang, P.R. China; University of Texas Medical Branch, United States of America

## Abstract

Severe fever with thrombocytopenia syndrome virus (SFTSV) infections have recently been found in rural regions of Zhejiang. A severe fever with thrombocytopenia syndrome (SFTS) surveillance and sero-epidemiological investigation was conducted in the districts with outbreaks. During the study period of 2011–2014, a total of 51 SFTSV infection cases were identified and the case fatality rate was 12% (6/51). Ninety two percent of the patients (47/51) were over 50 years of age, and 63% (32/51) of laboratory confirmed cases occurred from May to July. Nine percent (11/120) of the serum samples from local healthy people without symptoms were found to be positive for antibodies to the SFTS virus. SFTSV strains were isolated by culture using Vero, and the whole genomic sequences of two SFTSV strains (01 and Zhao) were sequenced and submitted to the GenBank. Homology analysis showed that the similarity of the target nucleocapsid gene from the SFTSV strains from different geographic areas was 94.2–100%. From the constructed phylogenetic tree, it was found that all the SFTSV strains diverged into two main clusters. Only the SFTSV strains from the Zhejiang (Daishan) region of China and the Yamaguchi, Miyazakj regions of Japan, were clustered into lineage II, consistent with both of these regions being isolated areas with similar geographic features. Two out of eight predicted linear B cell epitopes from the nucleocapsid protein showed mutations between the SFTSV strains of different clusters, but did not contribute to the binding ability of the specific SFTSV antibodies. This study confirmed that SFTSV has been circulating naturally and can cause a seasonal prevalence in Daishan, China. The results also suggest that the molecular characteristics of SFTSV are associated with the geographic region and all SFTSV strains can be divided into two genotypes.

## Introduction

Bunyaviruses constitute the largest family of viruses, including the genera *Hantavirus*, *Phlebovirus*, *Orthobunyavirus* and *Nairovirus*, which are pathogenic for humans [1]. The *Phlebovirus*, severe fever with thrombocytopenia syndrome virus (SFTSV), was first identified as the agent for severe fever with thrombocytopenia syndrome (SFTS) in rural areas of Hubei and Henan provinces in central China [2]. Subsequently, enhanced surveillance was implemented, and the epidemic areas of SFTSV infection from 2010 to 2013 were found to include at least fifteen Chinese provinces. SFTS cases have also been reported in South Korea, Japan, Mediterranean countries, and the United States [3].

The major clinical symptoms of SFTSV infection were reported to include fever, thrombocytopenia, gastrointestinal symptoms, and leukocytopenia, and there was an unusually high initial fatality rate of 30% [4]. The clinical course of SFTS patients could be divided into three major stages starting with fever, followed by multi-organ dysfunction and the convalescent stage [5]. As the clinical symptoms of SFTS can be confused with other infectious diseases, it is important to differentiate it from these diseases, especially haemorrhagic fever with renal syndrome (HFRS) caused by hantavirus and human anaplasmosis [6].

With the positive detection of SFTSV in *Haemaphysalis longicornis* ticks, ticks were considered to be the main vector for the transmission of SFTSV [7]. Sero-epidemiological studies showed that goats could also play a large role in transmission of SFTSV [8]. According to recent studies, SFTSV could also be transmitted from person to person through blood contact [9]. As the geographic area where SFTS is prevalent is large, SFTSV infection could be transmitted through various ways. Since the clinical symptoms of SFTS are severe and rate of case fatality is high, much attention therefore needs to be paid to controlling the spread of SFTSV.

SFTSV consists of a single-stranded negative-sense RNA genome, which includes three segments known as large (L), medium (M), and small (S) [2]. The L segment encodes the RNA-dependent RNA polymerase (RdRp), which is involved in viral transcription and replication. The segment M encodes the two viral envelope glycoproteins, G1 and G2, which are involved in immunogenicity and behave as neutralizing or protective epitopes. The S segment encodes two proteins, nucleocapsid protein (Np) and Non-structure protein (NS). Np facilitates viral RNA encapsulation and is responsible for the formation of RNA and protein complex [1].

It was reported that the large number of human recombinant MAbs derived from SFTS patients recognized the viral Np, suggesting that Np plays an important role during the human immune response to SFTSV infection. This critical epitope could thus provide a molecular basis for detection and diagnosis of SFTSV infection [10]–[11]. As the main structural protein, Np influences the serotypes of SFTSV, which are widely used for SFTSV antibody detection and for phylogenetic analysis [12]. It was also demonstrated that Np could suppress activation of IFN-γ and NF-κB signaling, which may dampen the innate immune response against SFTSV infection [13].

Our previous study identified for the first time SFTSV infections in isolated regions of China [14]. The results suggested that SFTSV in the climate and environment of these regions would show novel phenotypes and genotypes. In this study, we expanded the surveillance of SFTSV cases in Zhoushan, Zhejiang Province, for over three years and conducted sero-epidemiological studies. SFTSV were isolated, the whole genomes were sequenced, and Np genes were used for phylogenetic analysis. The characterization of epidemiological features of SFTS infections and genetic diversity analysis of SFTSV will contribute to our understanding of the prevalence of SFTSV infections.

## Materials and Methods

### Clinical samples collection

This study was approved by the ethics committee of Zhejiang Provincial Center for Disease Control and Prevention (CDC), China. Suspected SFTS cases were defined as manifesting sudden onset of febrile illness with body temperature ≥38°C, and leukopenia and/or thrombocytopenia without any known blood system disease or other known infectious or chronic diseases. With permission of the patients, serum samples were collected from the patients who were suspected to be infected with SFTSV, and all participants provided written informed consent.

### Viral RNA extraction and qRT-PCR identification

Viral RNA was extracted using the RNeasy Mini kit (Qiagen, USA) according to the manufacturer’s instructions. Briefly, 200 µl of the specimens were mixed with 600 µl of RLT buffer and 6 µl of β-mercaptoethanol and incubated for at least 1 min at room temperature. After the addition of 600 µl of cold 70% ethanol, the mixture was vortexed and applied to a spin-column. After the washing steps, RNA was eluted in 50 µl of RNase-free water.

As reported previously, specific Real-time qRT-PCR reactions were performed for the identification of the SFTSV infection using the One-Step Realtime qRT-PCR Kit (TaKaRa, Japan). Each reaction mixture consisted of 10 pmol of the primers F: 5′-TGGCCCTGGCTCTATAAACAT-3′, and R: 5′-AATGGCAGCCCAAATGAATC-3′; 5 pmol of the probe P: 5′-FAM-TCCAATGAYGCCAAGAAGTGGAA-BHQ1-3′; and 4 µl of template RNA in a final volume of 50 µl. Real-time qRT-PCR reactions were performed according to the instructions of the manufacturer.

### Virus isolation

Serum collected from the SFTS patients was used for virus isolation. Vero (Africa green monkey kidney cells) cell lines, which were maintained in MEM medium (Gibco, USA) supplemented with 2% fetal calf serum (FCS) (Gibco), 100 U/ml penicillin (Sigma, USA) and 100 mg/ml streptomycin (Sigma), in an atmosphere containing 5% CO_2_ at 37°C, were used for SFTSV isolation. The growth of the SFTSV in Vero cells was further determined by the qRT-PCR as described above.

### Whole genomic sequencing and phylogenetic analysis

RNeasy Mini Kit (Qiagen) was used for viral RNA extraction from the culture supernatants of different SFTSV isolates. The viral RNA was then used for the synthesis of cDNA through the application of RevertAid™ First strand cDNA Synthesis Kit (Thermo Fisher Scientific, USA). As described previously, using the primers designed according to the published sequences of the SFTSV, conventional PCR was applied for the sequencing of the whole genome of the SFTSV isolates. The terminal ends of viral RNA segments were determined with a RACE Kit (Invitrogen, USA). The whole genomic sequences were further confirmed through the application of the sequence-independent, single-primer amplification (SISPA) method [2]. The Gn, Gc, and Np gene DNA sequences of the SFTSV isolates were compared to the National Center for Biotechnology Information (NCBI) database through BLAST. Based on the sequences of the nucleocapsid genes, phylogenetic analysis was done using the Mega 5.10 software.

### B cell Epitope prediction and determination

The surface probability plot and hydrophilicity plot present in the Np from SFTSV was first analyzed with DNA STAR software. The Np sequences of different SFTSV strains were then submitted to the BepiPred 1.0 server for linear B cell epitopes prediction using a combination of a hidden Markov model and a propensity scale method (http://www.cbs.dtu.dk/services/BepiPred/).

The enzyme-linked immuno-sorbent assay (ELISA) was used to analyze the binding of different linear B cell epitopes to the SFTSV specific antibodies. Briefly, the predicted linear B cell epitopes were synthesized and the purity was determined through high performance liquid chromatography. Purified linear B cell epitopes were diluted to a concentration of 10 ng/ml in 0.05 M bicarbonate buffer (pH 9.6), and used to coat the 96-well EIA/RIA Stripwell immuno-plates (Corning, USA). Test serum was diluted serially in PBS containing 5% bovine serum albumin, and used as the primary antibody. HRP-labelled goat anti-rabbit-IgG diluted 1∶5000 (Jackson ImmunoResearch, USA) was used as the secondary antibody, and 1∶500-diluted serum from normal rabbit was used as the control. Cutoff values were determined as 2.1 times of the mean value from the negative control serum.

### Sero-epidemiological study

A sero-survey of healthy populations was also done in this study. Serum samples were collected from 112 healthy volunteers in the three villages where SFTSV infection cases were found. All volunteers denied having SFTS symptoms previously. Serum samples were tested for total antibodies (IgG and IgM) to SFTSV using a double-antigen sandwich ELISA kit, in which the recombinant nucleoprotein (Np) of SFTSV was used as the antigen [12]. Antigen slides were generated using SFTSV strains from Zhoushan. Indirect immuno-fluorescence assay (IFA) was performed for confirmation of the results from the ELISA [2].

## Results

### Surveillance of SFTSV and sero-epidemiological investigation

**Figure 1:**
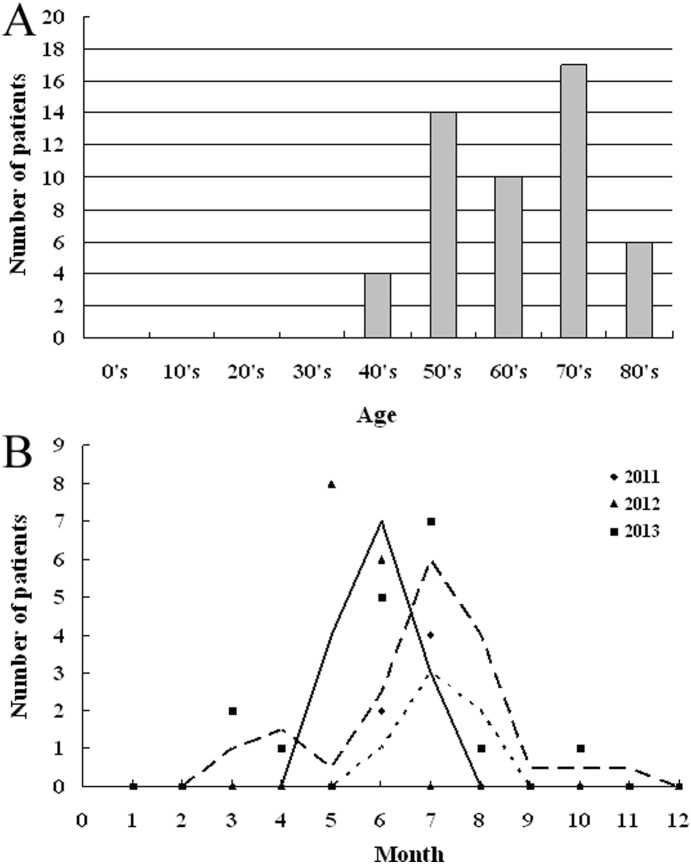
Surveillance results for SFTS in Daishan, Zhejiang Province. A: Age distribution of diagnosed SFTS patients in Daishan from 2011 to 2014. B: Seasonality distribution of identified SFTSV infection cases in Daishan from 2011 to 2013.

A total of fifty-one SFTSV infection cases were identified in Daishan during the study period of 2011–2014. The major clinical symptoms included fever, thrombocytopenia, and leukocytopenia. Among all the subjects with SFTS, 45% (23/51) were male and 55% (28/51) were female, and the rate of fatality was 12% (6/51). The ages of the patients ranged from 40–85 years, and 92% (47/51) of the patients were over 50 years of age. Thirty nine out of 51 (76%) patients were local farmers living in wooden and hilly areas. There were also patients who had the professions of teacher, fishermen, etc. Though the ticks were widely distributed in the patients’ living environment, only 14% (7/51) of the patients stated that they had been bitten by ticks before the onset of the disease. Through the analysis of the SFTS surveillance data from 2011–2013, most of the laboratory-confirmed cases 63% (32/51) happened from May to July. In addition, 4% (2/51) cases occurred in March, 2% (1/51) occurred in April, 2% (1/51) occurred in August, and 2% (1/51) occurred in October (Figure 1).

SFTSV can infect a variety of cells *in vitro*, including L929, Vero E6, Vero and DH82 cells. In this study, Vero cells were used for the SFTSV isolation. With detection by qRT-PCR, the SFTSV was found to be replicate efficiently in Vero cells, from which were obtained twelve SFTSV isolates. These isolates were further applied for genomic sequencing.

**Figure 2:**
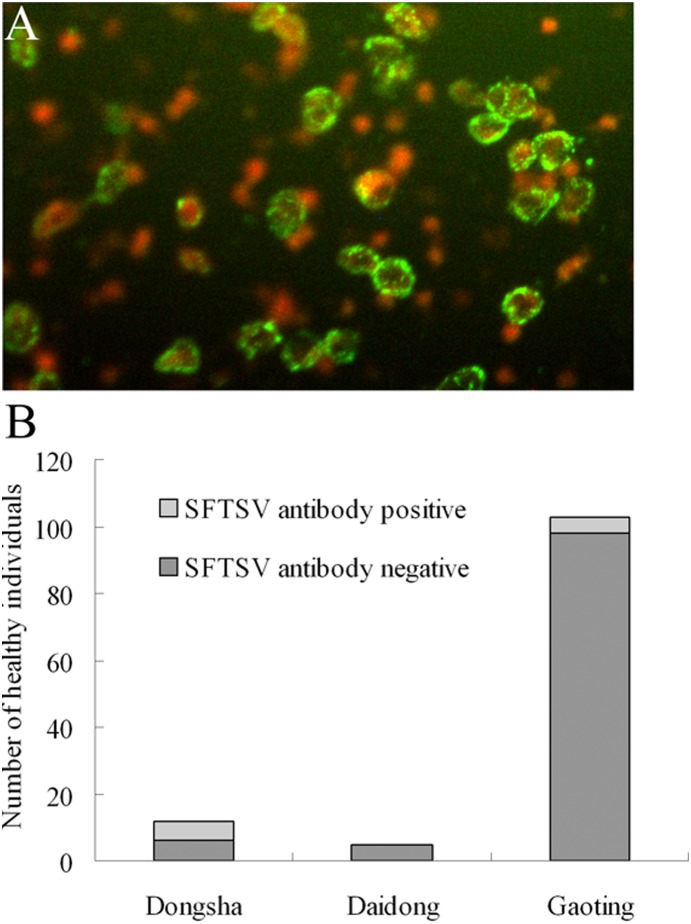
Sero-prevalence of SFTSV in healthy individuals from different villages of Daishan. A: IFA detection of IgG antibodies to SFTSV in healthy individuals. B: ELISA detection result of IgG and IgM antibodies to SFTSV in healthy individuals from different districts of Daishan.

The sero-prevalence of SFTSV among healthy people was conducted in three rural villages in Daishan. One hundred and twenty healthy volunteers from these villages were recruited. All the study participants were local residents, and blood samples were collected from all volunteers. The age and sex distribution of the study population may have been influenced by the ability to travel of people from the countryside to cities, or a lower participation rate among older and younger people. Serum samples were tested for total antibodies (IgG and IgM) to SFTSV using a double-antigen sandwich ELISA kit, and the detection results were confirmed by the IFA. Fifty % (6/12) were found to be positive for total antibodies to SFTS virus in Dongshan, no (0/5) positive samples were positive in Daidong, and the percentage of positive samples from Gaoting was 4.9% (5/103) (Figure 2).

### Sequencing and phylogenetic analysis

The whole genomes of the two SFTSV strains (01 and Zhao) from Daishan were successfully sequenced through next generation sequencing technology. As described previously, conventional PCR was also used for the confirmation of the sequencing results. The genomic sequences of these SFTSV strains were submitted to GenBank. The identities of the L, M, S segments for SFTSV strain Zhao were KF374682.1, KF374684.1, KF374683.1; and for SFTSV strain 01 they were KJ597825.1, KJ597824.1, KJ597823.1. The nucleocapsid genes of the SFTSV were used for phylogenetic analysis in this study.

The similarities of the target nucleocapsid genes from the SFTSV strains of different regions of China, including Anhui (GenBank No.: HQ141591.1), Henan (GenBank No.: HQ141597.1), Hubei (GenBank No.: HM745932.1), Jiangsu (GenBank No.: HQ141606.1), Liaoning (GenBank No.:HQ141609.1), Shandong (GenBank No.: HM802205.1), and Gangwon of South Korea (GenBank No.: KF358693.1), and Yamaguchi and Miyazakj of Japan (GenBank No.: AB817995.1, AB817997.1), were 94.2–100%.

**Figure 3:**
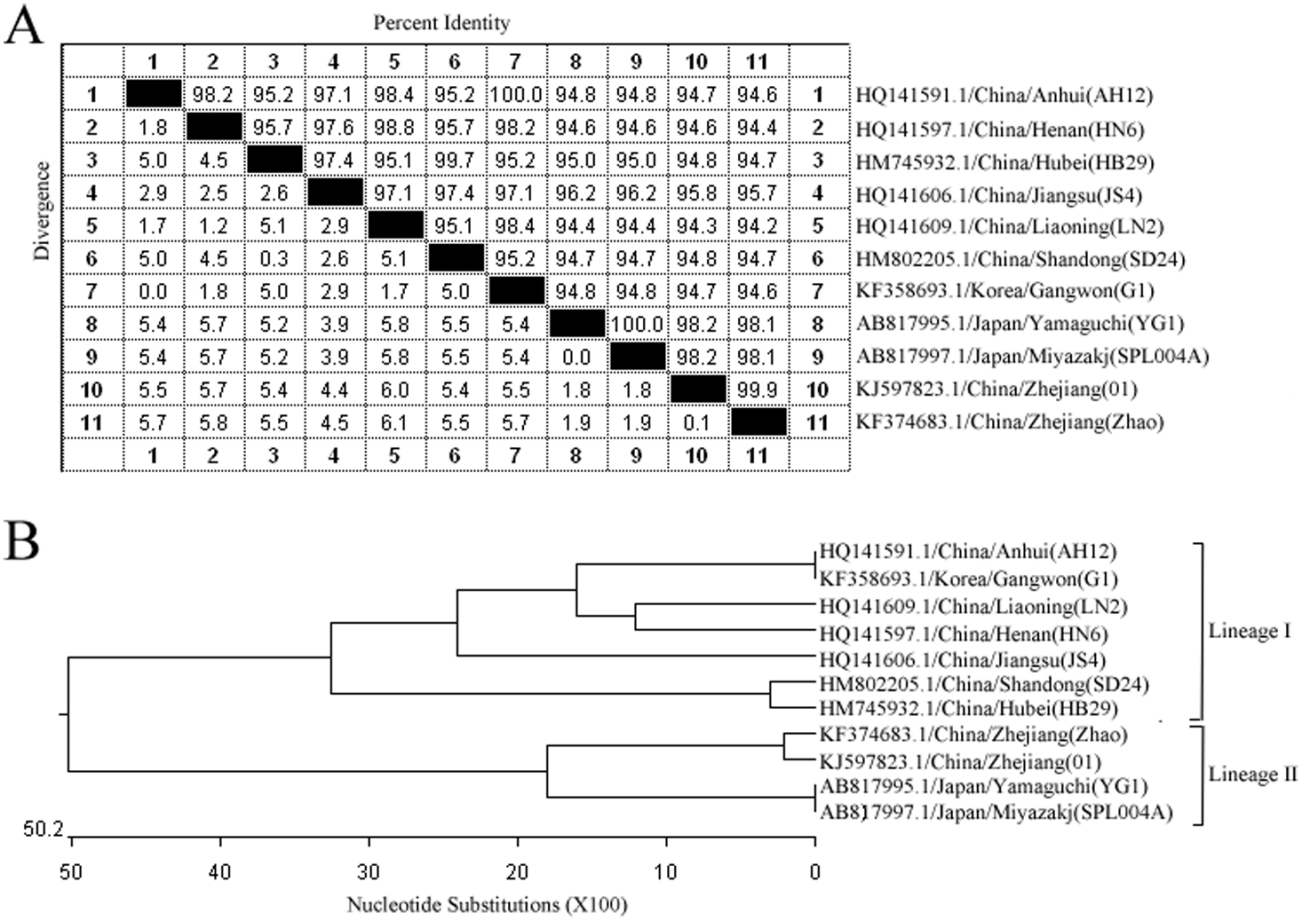
Homology comparison of the target nucleocapsid gene sequences from different SFTSV strains. A: The similarities of the target nucleocapsid gene sequences from the SFTSV strains of China (Henan, Liaoning, Shandong, Jiangsu, Hubei, Zhejiang and Anhui), Japan (Yamaguchi and Miyazakj), and South Korea (Gangwon) were 94.2–100%. B: Phylogenetic tree showed the phylogenetic positions of SFTSV strains in Zhejiang (Daishan), compared with other known SFTSV strains.

Based on the results from the sequence comparisons, a phylogenetic tree was generated. According to the nucleocapsid genes sequences, SFTSV can be divided into two lineages. Interestingly, SFTSV strains from the inland of China (Anhui, Henan, Hubei, Jiangsu, Liaoning, Shandong) and South Korea were clustered into lineage I. SFTSV strains from Zhejiang (Daishan), China, and Japan, both of which are isolated regions with a similar environment, were clustered into lineage II (Figure 3).

Amino acid sequences of Np from SFTSV of Zhejiang (Daishan) and Shandong were used for structure and antigenic analysis (Figure S1). It was predicted that there are eight linear B cell epitopes present in Np. Moreover, two linear B cell epitopes from these two SFTSV strains expressed mutations (Table 1). A structure analysis showed that the surface probability and antigenic index for Np was little affected. With the confirmation through detection by ELISA, it was found that all the ten linear B cell epitopes can bind to IgG specific for Np and SFTSV. Therefore, the mutations on the linear B cell epitopes have little effect on their ability to be recognized by specific antibodies.

**Table 1:** Prediction of linear B cell epitopes for nucleocapsid protein from different SFTSV strains.

GenBank Identity fordifferent Np	StartPosition	EndPosition	Linear B cellpeptide	Peptide length
HM802205.1	43	52	KETGGDDWV**K**	10
	155	163	TAGV**T**EATT	9
KF374683.1	43	52	KETGGDDWV**R**	10
	155	163	TAGV**S**EATT	9
HM802205.1 and KF374683.1	69	70	VK	2
	73	80	GKMSNSGS	8
	127	128	PV	2
	138	142	ENYPP	5
	186	196	RGASKTEVYNS	11
	224	237	ILGPDGVPSRAAEV	14

## Discussion

As a serious emerging infectious disease, severe fever with thrombocytopenia syndrome was first reported in rural areas of central China [2]. The characteristic symptoms of SFTS include fever, thrombocytopenia and leukocytopenia, and the initial case fatality rates were quite high. A cytokine-mediated inflammatory response, characterized by a cytokine and chemokine production imbalance, might be in part responsible for the disease progression of patients with SFTSV infection [15]–[16]. With the occurrence of SFTS cases in Zhejiang Province, enhanced surveillance was implemented.

As described previously, the first SFTSV infection case was found in Zhoushan [14]. However, the molecular and serological epidemiological features of SFTS in Zhoushan had not been characterized. We have now carried out the SFTS surveillance in Zhoushan for over three years, and the data analysis provides a clear picture for the seasonal prevalence and population susceptibility of SFTS.

It was found that SFTSV infections mainly happened from April to June every year, consistent with the growth period of local ticks, which are considered to be the vector for the transmission of SFTSV. As reported previously, the distribution of SFTS showed highly significant temporal and spatial heterogeneity in the Xinyang Region, with the majority of SFTS cases being among elderly farmers who resided in the southern and western parts of the region, mostly acquiring infection between May and July when the tick *H. longicornis* is highly active [17]. Thus, during these months, people who work in the outdoors should protect themselves from tick bites. However, most of the SFTS patients denied that they had been bitten by ticks before the onset of the illness. Through RT-PCR, we could not detect SFTSV RNA from the collected tick samples. We concluded that there were probably other vectors for SFTS transmission. Another study also reported that only 4.93% of *H. longicornis* and 0.613% of *R. microplus* populations were SFTSV RNA positive [7]. Therefore, in our future studies, we will attempt to measure the presence of SFTSV RNA in a larger numbers of ticks, while also paying attention to other potential vectors and host animals.

SFTSV infections have been reported in many countries, which were distributed in large areas of the world [3]. The environment of these epidemic areas is significantly different, which suggested that the molecular features of SFTSV from different areas might show large diversity. Phylogenetic analysis has already confirmed that SFTSV is a novel species of the genus *Phlebovirus*. We attempted to isolate SFTSV from Zhoushan, and performed whole genomic sequencing. Based on the Np gene sequences from different SFTSV strains, phylogenetic analysis results show that SFTSV from Zhoushan and Japan was distributed in one cluster, and SFTSV from inland areas such as Hubei and Henan was distributed in another cluster.

Previous phylogenetic analysis showed that Japanese SFTSV strains were closely related to Chinese SFTSV isolates, but formed an independent cluster, and that no geographical or chronological relationships existed between the Japanese and Chinese SFTSV strains [18]. However, our study suggested that the SFTSV strains could be divided into two genotypes based on the Np gene. In addition, SFTSV strains from Japan are related molecularly to SFTSV strains from isolated areas of China.

The Np gene was widely used as the target for SFTSV RNA detection, but the diversity of the sequences suggested that we should be careful about the mutations of this gene; otherwise the RT-PCR method would not be valid for the detection of all SFTSV strains. We also tried to determine if the diversity of the Np gene could contribute to new serotypes of SFTSV. Although the mutations of the Np gene resulted in a large diversity of linear B cell epitopes, the IFA and ELISA detection results showed that the mutations have no effect on the binding of Np to SFTSV-specific antibodies.

It was found that SFTSV readily infects humans involved in farming activities, as well as numerous domestic and wild animals, and that the overall sero-prevalence of SFTSV infection was 0.44% (11/2,510) in seven counties in Jiangsu Province [19]–[20]. Sero-prevalence studies were performed in the three villages where SFTSV cases were reported. The serology survey results showed that 9.2% of local healthy people were positive for SFTSV antibodies. These results indicate that SFTS infections are widespread in rural areas in the Zhoushan area, and subclinical SFTSV infections or mild form of SFTS illness might occur in humans.

SFTSV is circulating in Zhoushan, and the epidemiological features of infection are similar to infections in other epidemic areas. Phylogenetic analysis revealed that SFTSV strains from Zhoushan could be separated into a new genotype. Further research will be necessary to determine now SFTSV circulates in the wild environment.

## Supporting Information

Figure S1 **Structure analysis of nucleocapsid proteins from different SFTSV strains.** A: Surface probability and antigenic index prediction results for nucleocapsid protein from SFTSV strain from an inland region of China (Shandong). B: Surface probability and antigenic index prediction results for nucleocapsid protein of SFTSV strains from isolated area of China (Daishan of Zhejiang).
(TIF)
