# Supplementary figures and images for: Characterization of Severe Fever with Thrombocytopenia Syndrome in Rural Regions of Zhejiang, China
Source: PLoS One. 2014 Oct 30;9(10):e111127. doi: 10.1371/journal.pone.0111127 (PMC4214719; doi:10.1371/journal.pone.0111127)

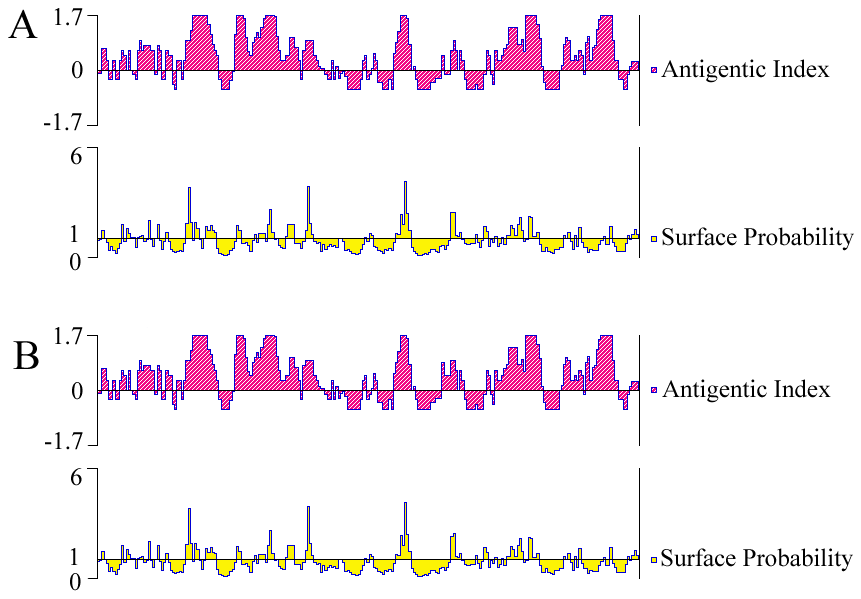

Supplement: Figure S1 — Structure analysis of nucleocapsid proteins from different SFTSV strains. A: Surface probability and antigenic index prediction results for nucleocapsid protein from SFTSV strain from an inland region of China (Shandong). B: Surface probability and antigenic index prediction results for nucleocapsid protein of SFTSV strains from isolated area of China (Daishan of Zhejiang). (TIF) [file pone.0111127.s001.tif]
